# Minocycline Administration Does Not Have an Effect on Retinal Ganglion Cell Survival in a Murine Model of Ocular Hypertension

**DOI:** 10.14336/AD.2024.0224

**Published:** 2024-10-01

**Authors:** María del Cielo Sánchez-Migallón, Johnny Di Pierdomenico, Alejandro Gallego-Ortega, Diego García-Ayuso, Manuel Vidal-Sanz, Marta Agudo-Barriuso, Francisco J. Valiente-Soriano

**Affiliations:** Departamento de Oftalmología, Universidad de Murcia e Instituto Murciano de Investigación Biosanitaria (IMIB)-Pascual Parrilla. Campus de CC de la Salud, El Palmar, Murcia, Spain.

**Keywords:** OHT, apoptosis, caspase 3, RGC, microglia, minocycline

## Abstract

This study aims to investigate two key aspects in a mouse model of ocular hypertension (OHT): first, the time course of retinal ganglion cell (RGC) death and the parallel activation of caspase-3 (a-Casp3+ cells) to narrow the therapeutic window; and second, the effect of caspase-3 and microglia inhibition by minocycline on RGC rescue in this model. RGC loss after OHT induction was significant at day 7 and progressed to 30 days. However, anatomical RGC death was preceded by significant Casp3 activation on day 3. Microglial inhibition by minocycline did not alter the course of OHT or rescue RGCs but resulted in a decrease in a-Casp3+ cells and phagocytic and total microglia. Therefore, RGC death commitment occurs earlier than their loss of Brn3a expression, microglial cells do not exacerbate RGC loss, and while this death is primarily apoptotic, apoptosis inhibition does not rescue RGCs, suggesting that alternative death pathways play a role in glaucomatous injury.

## INTRODUCTION

Glaucomatous optic neuropathy (GON) is a pathology that causes continuous retinal ganglion cell (RGC) degeneration [1, 2]. There are several risk factors for GON, including age, family history, race and increased intraocular pressure (IOP). Of these, only ocular hypertension (OHT) is modifiable, and the use of pharmacological or surgical strategies to lower IOP is currently the only therapeutic option for these patients, although in some cases this treatment is ineffective [3]. Therefore, alternative pathways of RGC protection are being explored through the administration of neuroprotective agents that act in parallel with IOP lowering and regulation.

For this purpose, animal models of OHT have been developed to induce RGC degeneration analogous to that observed in GON patients [4-11]. These models are suitable for searching neuroprotective therapies for RGCs by blocking death pathways [12-14], or by activating pro-survival pathways [5, 15-21].

To develop an effective therapeutic strategy for RGCs, it is necessary to understand the evolution of their degeneration from the onset of injury. The model of OHT we use in this study is induced by diode laser photocoagulation of the perilimbal and episcleral veins in the adult Swiss albino mouse, which alters the drainage of the aqueous humour producing a transient elevation of IOP which in turn results in a significant and sectorial death of RGCs, causing the loss of 60-70% of their population by 7-8 days without further death up to at least 63 days [6, 22]. Death of RGCs after OHT is, at least in part, induced by apoptosis [23-25]. Indeed, there is a peak of active caspase-3 (a-Casp3) expression during the first days after IOP elevation [24] that precedes the loss of RGCs [26].

Concomitant to RGC death, retinal glia, both macro and microglial cells (MCs), react to the injury and activate as early as 24 hours after OHT induction [27-32]. Furthermore, MCs change to more activated/amoeboid morphology with the presence of phagocytic MCs (PMCs) [29, 33]. PMCs play a decisive role in retinal lesions because they phagocytose the bodies of dead neurons cleaning the tissue to restore homeostasis. Thus, there is an inverse correlation between the loss of RGCs and the appearance of PMCs [33]. Although this is a vital task, over-activation of MCs can have an undesirable effect by causing further degeneration modulated by the induction of chronic neuroinflammation [34-36].

The aims of the present work were, first, to define the therapeutic window in this model by correlating RGC death with the expression of a-Casp3; second, to investigate the effect of administration of minocycline, a tetracycline antibiotic that inhibits microglia [35, 37-42] and a-Casp3 [40, 43, 44], and third, to study the dynamics of MCs and PMCs in this model with or without minocycline treatment.

## MATERIALS AND METHODS

### Animal handling

To carry out these experiments, we used 104 adult male Swiss albino mice (?30 g) provided by Charles River (Barcelona, Spain) and housed in temperature-controlled rooms with 12h light/dark cycles (from 8.00 am to 8.00 pm) and with food and water supplied ad libitum in the animal facilities of the University of Murcia (UM, Spain). Animal care and experimental procedures were conducted in accordance with the Spanish and European Union for the use of animals in research (2010/63/EU directive) and the ARRIVE guidelines, and the ARVO statement for the use of animals in ophthalmic and vision research and were approved by the UM Ethical and Animal Studies Committee (Codes: A13171103, A13170110, and A13170111). All invasive procedures were performed under anaesthesia by intraperitoneal administration of a mixture of xylazine (10 mg/kg body weight; Rompun®; Bayer, Kiel, Germany) and ketamine (60 mg/kg body weight; Ketolar®; Pfizer, Alcobendas, Madrid, Spain). In addition, during and after the interventions the eyes were covered with an ointment (Tobrex; Alcon S. A., Barcelona, Spain) to prevent corneal desiccation and a subcutaneous injection of buprenorphine (0.1 mg/kg; Buprex, Buprenorphine 0.3 mg/mL; Schering-Plough, Madrid, Spain) was administered as analgesia.

### Experimental design

To assess the temporal course of RGC death and the expression of a-Casp3 after OHT, the intraocular pressure was raised in 50 experimental mice that were sacrificed 3, 4, 5, 7, 15 or 30 days later (n=8/time point). Two intact mice (4 retinas) were used as controls. To analyze whether minocycline treatment altered the course of RGC loss and the expression of a-Casp3, mice were intraperitoneally administered minocycline hydrochloride (14 mice) or vehicle (saline, 14 mice) daily starting the day before OHT induction and were sacrificed 4 or 15 days after OHT (n=7/treatment/time point). RGCs were retrogradely labelled from the superior colliculi (SCi) with hydroxystilbamidine methanesulfonate (OHSt) one week before OHT induction in 24 animals to study the response of MCs (Iba1^+^MCs) and PMCs (OHSt+Iba1+) to OHT with or without minocycline treatment. One day before OHT induction 12 mice were treated with minocycline and 12 with vehicle as in the previous group, and the animals were sacrificed 4 or 15 days after OHT induction (n=6/treatment/time point). In addition, 2 mice traced with OHSt from SCi (4 retinas) were used as controls. All retinas were dissected as flatmounts [45].

### Ocular Hypertension Induction

The OHT model was carried out in the left eyes of the experimental mice, following the same protocol as previously described [6, 30, 46-49]. In summary, a single session of diode laser shots (Viridis Ophthalmic Photocoagulator-532 nm laser, Quantel Medical, Clermont-Ferrand, France) directly targeted the perilimbal and episcleral veins for complete photocoagulation (360°, 65-70 shots per eye, spot size of 50-100 µm, power of 0.3 W and duration of 0.5 s). Prior to surgery, the pupils were dilated with 1% Tropicamide (Colircusi tropicamide 1%; Alcon-Cusí, S.A., Barcelona, Spain).

The IOP of both eyes was measured in anaesthetised mice between 9.00 and 10.00 am, to avoid circadian variations in IOP, using a mouse-adapted rebound tonometer (Tono-Lab; Tiolat, OY, Helsinki, Finland) as described previously [5, 6, 11]. Readings were obtained before and 1, 2, 5, 7, 15 and 30 days after OHT induction. Mean IOP per animal/d is the average of 6 independent measurements.

### RGC tracing

To trace the whole population of RGCs, one week prior to OHT induction, OHSt (Molecular Probes, Leiden, The Netherlands) diluted 10% in 10% dimethyl-sulfoxide/saline, was applied to both SCi, the major retinorecipient areas in mice [50, 51] following standard protocols in our group, as described previously [11, 45, 52, 53]. After retinal injury, the PMCs that phagocytose the RGCs' bodies are transcellularly labelled (OHSt+Iba1+) and are easily identifiable [29, 33]. However, this technique does not discriminate phagocytic microglia from infiltrating monocyte-derived macrophages (MdMs), so these cells are termed PMCs/MdMs.

### Minocycline administration

An intraperitoneal injection of 45 mg/kg minocycline (M9511; Merck-Millipore, Alcobendas, Madrid, Spain) dissolved in saline was administered daily starting the day before OHT induction, as previously described [35, 39, 41, 54]. Vehicle-matched groups were performed in parallel.

### Tissue processing

All mice were sacrificed by intraperitoneal lethal injection of pentobarbital (Vetoquinol Dolethal, Especialidades Veterinarias, S.A., Alcobendas, Madrid, Spain) and perfused transcardially with saline and 4% paraformaldehyde (PFA) in 0.1 M phosphate buffer (pH 7.4). Eyes were enucleated and postfixed for an additional hour in 4% PFA. Retinas were dissected and, to maintain retinal orientation, four cuts were made to mark superior, inferior, temporal and nasal positions, with the superior cut being the deepest, following previous protocols [26, 55].

### Immunohistofluorescence

Flat mounted retinas were immunodetected as described [45]. Immunodetection of Brain-Specific Homeobox/POU Domain Protein 3A (Brn3a) was used to identify vision-forming RGCs [56], a-caspase 3 to detect apoptotic cells [26], and ionised calcium-binding adapter molecule 1 (Iba1) for microglial cells [29]. Primary antibodies were goat α-Brn3a (1:500; C-20, Cat# sc-31984, RRID: AB_ 2167511, Santa Cruz Biotechnologies Heidelberg, Germany), rabbit α-cleaved-caspase3 (1:500; [Asp175], Cat#9664 (also 9664P), RRID:AB_2070042, Cell Signaling, Werfen/Izasa, Barcelona, Spain) and rabbit α-Iba1 (ionised calcium-binding adapter molecule 1 (1:500; Cat# ab178846, RRID:AB_2636859, Abcam, Cambridge, United Kingdom). Secondary detection (1:500) was carried out with donkey α -goat Alexa 488 (Molecular Probes, now part of Thermofisher, Cat# A-11055 (also A11055), RRID:AB_2534102), donkey α-rabbit Alexa 594 (Molecular Probes, now part of Thermofisher Cat# A-21207 (also A21207), RRID:AB_141637) and donkey α-rabbit Alexa 488 (Molecular Probes now part of thermofisher Cat# A-21206 (also A21206), RRID:AB_2535792) antibodies, respectively. Antibodies validated in the RRID database (https://www.rrids.org/) for research resource identification.

### Image analysis

All flat-mounted retinas were analysed and photographed under a microscope (Axioscop 2 Plus; Zeiss Mikroskopie, Jena, Germany) equipped with fluorescence as previously described [57] and 154 frames were acquired from each retina to reconstruct the entire retinal photomontage in a raster scan pattern (×20) contiguously, side-by-side with no overlap or spacing between images. Images of Brn3a+RGCs, a-Casp3+cells, Iba1+MCs and PMCs/MdMs were always taken focusing on the RGC layer (RGCL).

### Quantification and topographical distribution

The total population of Brn3a+RGCs was automatically quantified, and their distribution illustrated by isodensity maps as previously described [55, 58]. Isodensity maps show the density of RGCs/mm2 with a colour scale that goes from 0 RGCs/mm2 (purple) to ≥5700 RGCs/mm2 (red). All a-Casp3+cells, Iba1+MCs and OHSt+Iba1+ PMCs/MdMs with the focus on the RGCL-fibre layer were manually dotted on the retinal photomontages using Adobe Photoshop CS8.0.1 graphic editing software (Adobe Systems, Inc., San Jose, CA, USA) and the dots were automatically quantified using Image Pro Plus software (IPP 5.1 for Windows®; Media Cybernetics, Silver Spring, MD, USA). Their distribution was visualised using the nearest neighbour algorithm [55, 59] that shows the number of neighbours around a given cell in a radius of 0.2 mm with a colour scale that goes from 0-2 neighbours (purple) to ≥14 neighbours (red). For a-Casp3+cells, 0-8 neighbours (purple) to ≥32 neighbours (red) for Iba1+MCs and 0-4 neighbours (purple) to ≥18 neighbours (red) for OHSt+Iba1+ PMCs/MdMs.

### Statistical analysis

All graphs and the statistical analyses were done with GraphPad Prism 9 (GraphPad San Diego, USA). Data are presented as the mean ± standard deviation (SD). IOP values passed the normality test (Shapiro-Wilk) and were compared with one-way ANOVA. Cell number data were non-parametric and were compared with Kruskal-Wallis (more than 2 groups) or Mann-Whitney (two groups) tests. Comparisons were considered significant when p≤0.05. The statistical tests are detailed in figure legends and results.

## RESULTS

### Intraocular pressure increase

**Figure 1.**
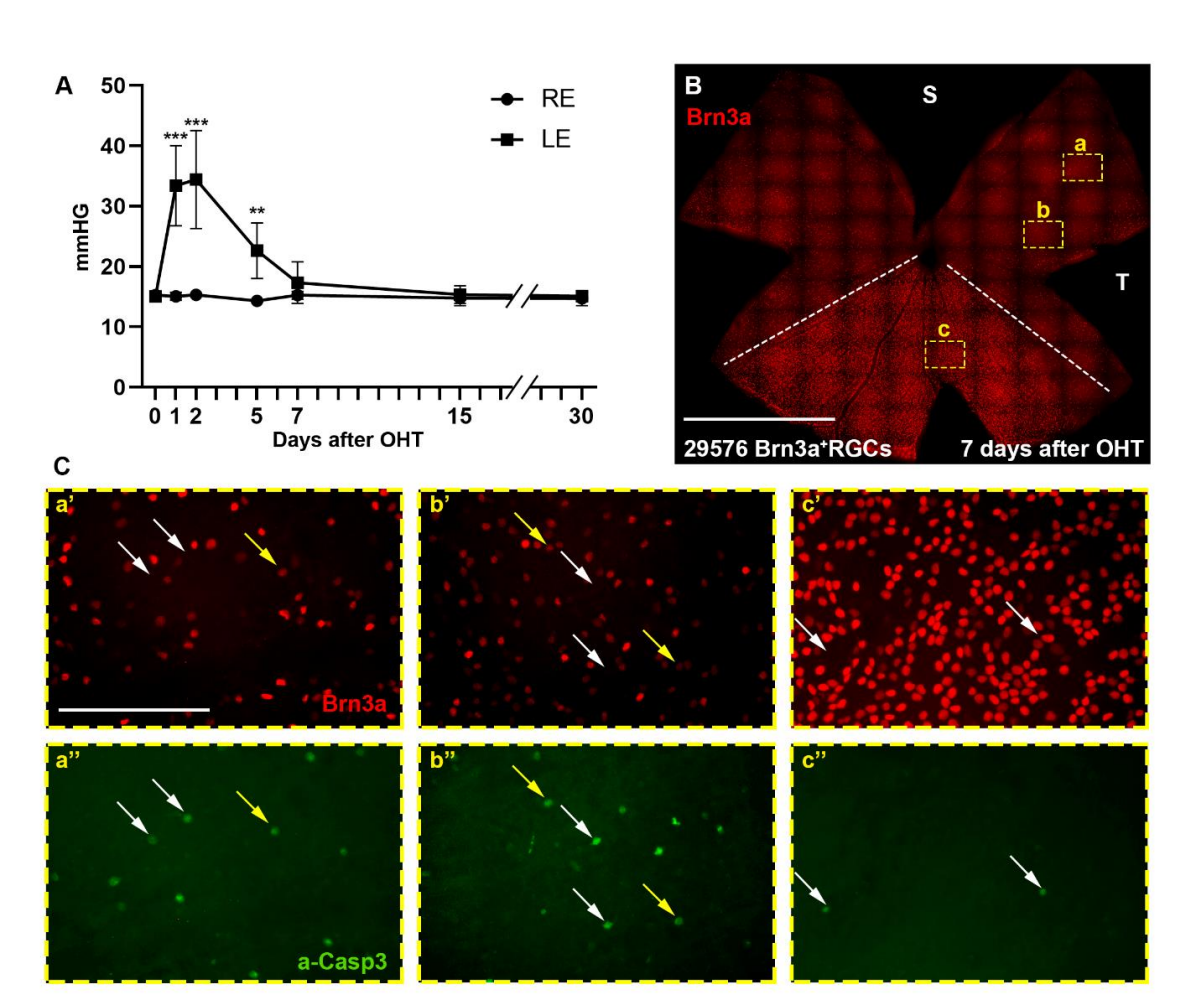
IOP evolution and Brn3a^+^RGCs and a-Casp3^+^cells after OHT induction. (A) X, Y graph (time, IOP) showing the mean IOP values (mmHg) ± SD in experimental left eyes (LE) and control right eyes (RE) before (day 0) and at 1, 2, 5, 7, 15 and 30 days after OHT induction. (0, 1- and 2-days n=48, 5 days n=32, 7 days n=24, 15 days n=16, 30 days n=8). * Significant vs. day 0; ***p<0.0001, **, p<0.01; one-way ANOVA). (B) Flat mounted retina analysed 7 days after OHT showing the sectorial loss of Brn3a^+^RGCs, which is more accentuated in the superior retina than in the ventral retina. The dashed white line demarcates both areas. (C) Magnifications of a-c squares in A, showing Brn3a^+^RGCs (a'-c') and a-Casp3^+^cells (a''-c'') in two areas of high (a, b) and one area of low RGC death (c). A higher number of a-Casp3^+^cells was observed in the regions of higher damage (a'', b'') than in the regions where the loss of RGCs is less pronounced (c''). In addition, it can be observed that some a-Casp3^+^cells are Brn3a^+^ (yellow arrows in a-c''), indicating that the apoptotic process is initiating, while other cells are a-Casp3^+^Brn3a^-^, indicating apoptosis is more advanced, or other Brn3a^-^ neurons undergoing apoptotic process. S: superior. T: temporal. Scale bar = 500µm.

Figure 1A shows the IOP values in the left (hypertensive) and right (contralateral) eyes before (day 0) and from 1 to 30 days after OHT induction. These values are similar to those reported in previous studies [6, 48] and show some variability between animals, especially in the first days after OHT induction (Supplementary Fig. 1). Twenty-four hours after the intervention, the IOP of the experimental eyes increased to 33.4±6.6 mmHg, 2.2-fold higher than its baseline value (15.1±0.6 mmHg; p<0.0001, one-way ANOVA). The IOP remained elevated at 2 days, decreased at 5 days and was not significantly different from baseline at 7 days (17.3±3.4 mmHg p=0.99, one-way ANOVA). Thereafter, values remained stable and within normal values (Fig. 1A).

**Figure 2.**
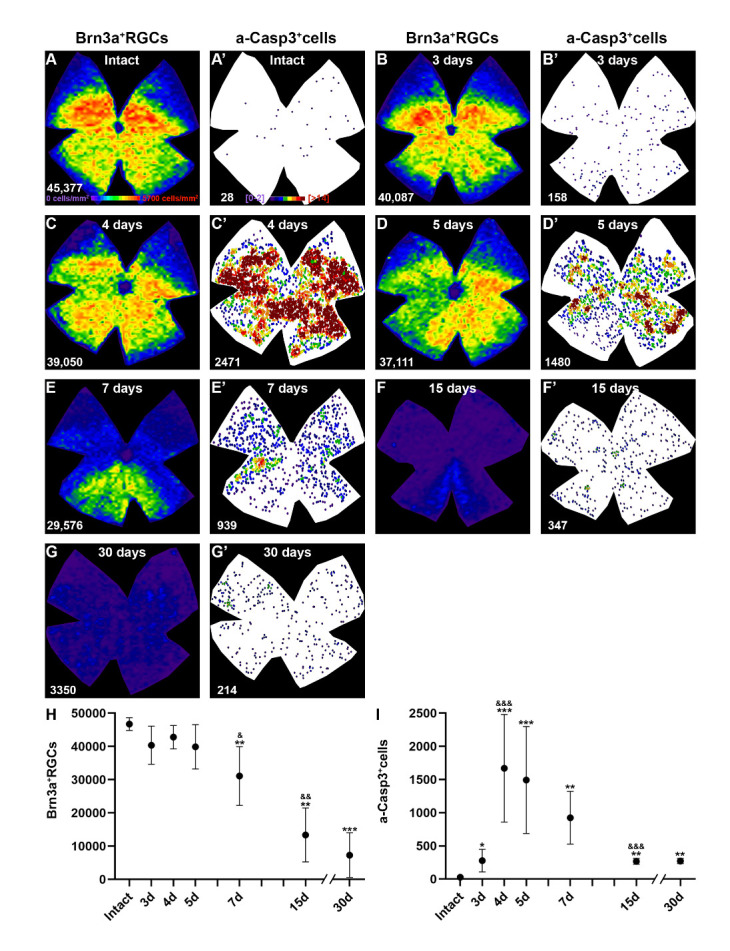
Course of RGC death and caspase 3 activation after ocular hypertension. Paired isodensity (A-G) and neighbour maps (A’-G’) showing the distribution of Brn3a^+^RGCs (A-G) and a-Casp3^+^cells (A’-G’) in intact retinas (A, A’) and retinas analyzed at 3 (B, B'), 4 (C, C'), 5 (D, D'), 7 (E, E'), 15 (F, F') or 30 days (G, G') after OHT. Both cell populations from the same retina are shown for each group. Isodensity maps colour scale ranges from 0-500 RGCs/mm^2^ (purple) to ≥5700 RGCs/mm^2^ (red). Neighbour maps colour scale ranges from 0-2 neighbours (purple) to ≥14 neighbours (red) within a radius of 0.2 mm. (H) X, Y graph (time T, number of Brn3a^+^RGCs) showing the mean total number of Brn3a^+^RGCs ±SD in intact retinas and retinas analyzed at 3, 4, 5, 7, 15 or 30 days after OHT. (I) X, Y graph (time, number of a-Casp3^+^cells) showing the mean total number of a-Casp3^+^cells ±SD in the same retinas as H. *Statistical significance compared to intact retinas. ^&^Statistical significance compared to the previous time point. One symbol p<0.05, two symbols p<0.01, and three symbols p<0.001, Mann-Whitney test).

### RGC loss and expression of a-Casp3 in the RGC layer after OHT

In the OHT model, RGC degeneration typically occurs in sectors with their apex pointing towards the optic nerve [6, 11]. Consistent with this, as shown in figures 1B and 2, some sectors, particularly those of the dorsal retina, showed an apparent loss of RGCs, whereas in others, normally located in the ventral retina, RGC survival was higher.

RGCs death followed the apoptotic pathway as shown by the expression of a-Casp3 in the RGCL (Fig. 1C), similar to that documented in optic nerve transection (ONT) or crush (ONC) models [26]. Indeed, as shown in figure 1C in one retina analysed 7 days after OHT, more a-Casp3+cells were observed in regions with higher RGC death (Fig. 1B, C a-b'') than in regions with higher RGC survival (Fig. 1B, C c-c’’). Specifically, there were a-Casp3+ Brn3a+RGCs (yellow arrows in Fig. 1C a'-b''), which are RGCs in the early stages of apoptosis, and a-Casp3+cells Brn3a- (white arrows in Fig. 1C a'-c''), which are RGCs in a more advanced apoptotic process in which Brn3a expression has already been downregulated, as previously described [26, 28]. Brn3a-Casp3+cells, they could also be such as melanopsin-expressing RGCs [55] or ON-alpha RGCs [60] which do not express Brn3a but are undergoing apoptosis.

### Quantitative and topographical analysis of the temporal degeneration of RGCs and caspase 3 activation

The topographical distribution of RGCs in the intact retina (46,712±1920 Brn3a+RGCs per retina, n=4) showed the highest cell density in the dorso-central region and the lowest in the periphery (Fig. 2A,H), in agreement with the distribution reported in previous studies [11, 58]. The RGC population remained within normal values for up to 5 days after OHT. At this time point RGC numbers were lower than those in intact retinas, but their loss did not reach statistical significance, although sectors of diminished RGC density were already visible in the isodensity maps (Fig. 2D, H).

A significant decrease of RGCs was observed at 7 days (31,088±8829 Brn3a+RGCs; n=8, p=0.008, Mann-Whitney test) (Fig. 2H) when the sectorial RGC loss was evident (Fig. 2B-E). Thereafter, the population of RGCs gradually decreased until day 15 (13,373±8104 Brn3a+RGCs). After this time, RGC loss did not progress significantly at least up to 30 days (7287±6737 Brn3a+RGCs, n=8) (Fig. 2A-H).

In these same retinas, we quantified and plotted the topography of the total number of a-Casp3+cells found in the RGCL (Fig. 2A’-G’, I). There was a significant increase in a-Casp3+cells 3 days after OHT (279±169 n=8, p=0.0061, Mann-Whitney test) compared to intact retinas (22±11, n=8). Their number peaked at 4 days (1671±810 a-Casp3+cells, n=8, p=0.0002, Mann-Whitney test), was maintained at 5 days and started to decrease progressively stabilising at 15 and 30 days (269±44 and 272±39 a-Casp3+cells, respectively) with values significantly higher than in intact retinas (p=0.0002 and p=0.0009, respectively, Mann-Whitney test) and similar to 3 days (Fig 2B'-G’, I).

In terms of a-Casp3+cell distribution, a diffuse pattern was observed throughout the retina at the earlier (3 days) and later (15-30 days) time points (Fig. 2, compare Brn3a+RGCs isodensity maps with their corresponding a-Casp3+cells neighbour maps). Interestingly, even though at 4 and 5 days the loss of RGCs was not quantitatively significant, there were areas of density loss (observed by the loss of warm colours in the isodensity maps Fig. 2C, D) and in these areas there was a higher number of a-Casp3+cells. This co-topography was more obvious at 7 days (Fig. 2E-E’).

**Figure 3.**
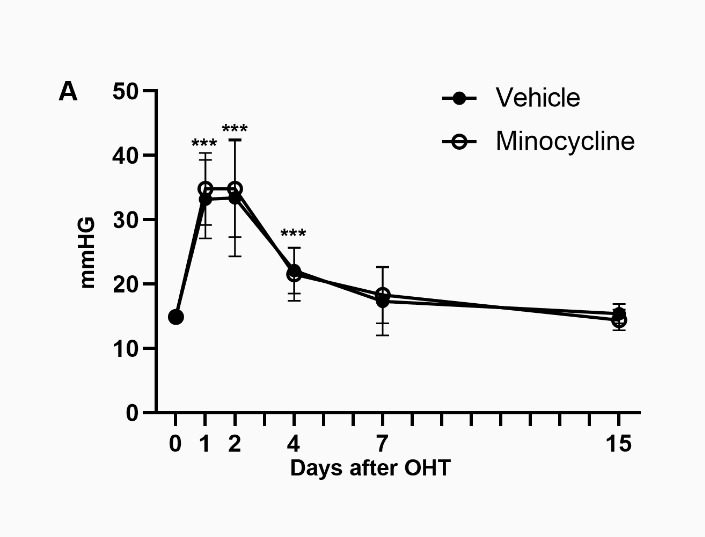
Microglial inhibition by minocycline treatment does not alter the IOP evolution in OHT-eyes. X, Y graph (time, IOP) showing the mean IOP values (mmHg) ± SD in experimental eyes of minocycline- and vehicle-treated animals before and at 1, 2, 5, 7, 15 and 30 days after OHT induction (0-, 1-, 2- and 4 days n=28, 7- and 15 days n=14) (***p<0.001 compared to day 0, one-way ANOVA).

### Effect of daily minocycline administration on the evolution of RGCs loss and a-Casp3 expression after OHT

Daily intraperitoneal minocycline or vehicle administration had no effect on IOP evolution after OHT (Fig. 3) which followed the same course as that of the experimental eyes of untreated animals shown in Figure 1A (p=0.7656, one-way ANOVA).

The effect of daily minocycline administration on RGC survival, appearance of a-Casp3+cells and dynamics of MCs and PMCs/MdMs (see below) was investigated at 4 days after OHT, when the number of a-Casp3+cells peaked (Fig. 2I) and at 15 days after OHT, when the loss of Brn3a+RGCs stabilised (Fig. 2H).

**Figure 4.**
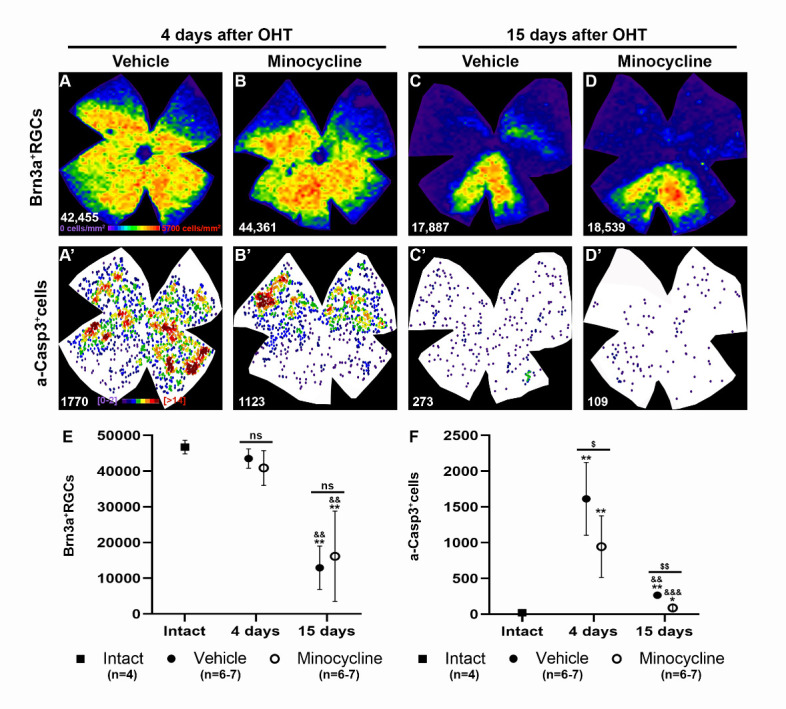
Minocycline treatment does not ameliorate RGC loss after OHT but decreases the expression of a-Casp3. (A-D) Isodensity maps showing the topographical distribution of Brn3a^+^RGCs in hypertensive retinas treated with minocycline (B, D) or vehicle (A, C) and analysed at 4 (A, B) or 15 days (C, D) after OHT. A’-D’: Neighbour maps showing the topographical distribution of a-Casp3+Cells in the same retinas as A-D. Isodensity maps colour scale ranges from 0-500 RGCs/mm^2^ (purple) to ≥5700 RGCs/mm^2^ (red). Neighbour maps colour scale goes from 0-2 neighbours (purple) to ≥14 neighbours (red) within a radius of 0.2 mm. (E) Graph showing the mean total number of Brn3a^+^RGCs ± SD in intact (n=4) or hypertensive retinas treated with minocycline (n=5-6) or vehicle (n=6) and analyzed at 4 or 15 days after OHT. (F) Graph showing the mean total number of a-Casp3^+^cells ± SD in the same retinas as E. *Statistically significant compared to intact retinas. ^&^Statistically significant compared to the previous time point within groups. ^$^Statistical significance between treated and untreated groups. One symbol p<0.05, two symbols p<0.01, and three symbols p<0.001, Mann-Whitney test).

Minocycline treatment had no effect on RGC survival (16,161±12,650 Brn3a+RGCs, (n=6)) 15 days after OHT compared with vehicle treatment (12,919±6085 Brn3a+RGCs (n=7), p=0.9015, Mann-Whitney test) (Fig. 4A-D, E). However, minocycline administration significantly decreased the number of a-Casp3+cells at 4 and 15 days in these retinas (945±431 and 89±41 a-Casp3+cells, p=0.048, and p=0.0012, respectively, Mann-Whitney test) (Fig. 4A’-D’, F). Irrespective of the treatment, a-Casp3+cells were found delimiting the sectors of RGC death at 4 days and throughout the retina at 15 days, as observed in the previous analyses (Fig. 1 and 2).

### Effect of daily minocycline administration on the dynamics of microglial cells and PMCs in the retinal ganglion cell layer (RGCL) after OHT

**Figure 5.**
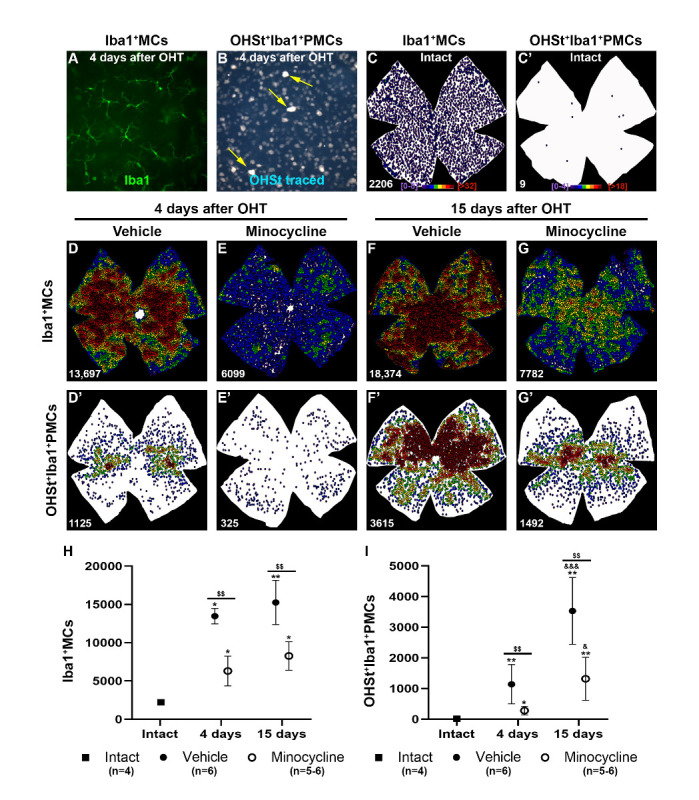
Minocycline treatment decreases the number Iba1^+^MCs and OHSt^+^Iba1^+^PMCs in hypertensive retinas. (A-B) Retinal magnifications showing Iba1^+^MCs (A) and OHSt^+^Iba1^+^PMCs/MdMs (yellow arrows in B) in the same RGCL area 4 days after OHT. (C-G) Neighbor maps showing the distribution of Iba1^+^MCs in intact (C) vehicle- (D, E) or minocycline- (F, G) treated retinas analyzed at 4 (D, E) or 15 days (F, G) after OHT. C’-G’ Neighbor maps showing the distribution of OHSt^+^Iba1^+^PMCs/MdMs the same retinas as C-G. Neigbour maps colour scale for Iba1^+^MCs goes from 0-8 neighbours (purple) to ≥32 neighbours (red) within a radius of 0.2 mm and for OHSt^+^Iba1^+^ PMCs/MdMs from 0-4 neighbours (purple) to ≥18 neighbours (red) within the same radius. (H, I) Graphs showing the mean total number ± SD of Iba1^+^MCs (H) and OHSt^+^Iba1^+^ PMCs/MdMs (I) in intact (n=4) or hypertensive retinas treated with minocycline (n=5-6) or vehicle (n=6) and analyzed at 4 or 15 days after OHT. *Statistically significant compared to intact retinas. ^&^ Statistically significant compared to previous time point within groups. ^$^ Statistical significance between treated and untreated groups. One symbol p<0.05, two symbols p<0.01, and three symbols p<0.001, Mann-Whitney test).

Next, we quantified and mapped the total number of Iba1+MCs, and of OHSt+Iba1+ PMCs/MdMs (Fig. 5). The former correspond to all Iba1+cells and the latter to PMCs/MdMs. Thus, to carry out this analysis we first traced RGCs from the superior colliculi with OHSt, a retrogradely transported fluorescent tracer. When RGCs die, microglial cells phagocytose their somas, which are filled with the tracer. As a result, the microglial cells become transcellularly labelled as the tracer accumulates in their phagolysosomes. PMCs/MdMs can be identified by their morphology, which differs from that of traced RGCs, but to ensure that they were indeed PMCs/MdMs, we quantified Iba1+OHSt+ cells (Fig. 5A-B ) [29].

In intact retinas there was a mean±SD of 2231±181 Iba1+MCs (n=4) distributed homogeneously in the RGCL (Fig. 5C, H). Their number significantly increased in OHT-retinas from vehicle and minocycline treated animals at 4 (13,468±1015 Iba1+MCs (n=5) and 6306±1955 Iba1+MCs (n=5), respectively) and 15 days (15,260±2884 Iba1+MCs (n=6), 8274±1862 Iba1+MCs (n=6), respectively). However, at both time points minocycline treatment greatly reduced the number of MCs (p=0.0079 for both times, Mann-Whitney test) (Fig. 5D-G, H).

At 4 and 15 days after OHT, microglial cells were found throughout the retina with higher densities in the central medial areas (Fig. 5E, G). In minocycline-treated retinas, microglial cell density was lower than in vehicle-treated retinas, in accordance with the quantitative data, and their distribution was homogeneous at 4 days, whereas at 15 days their density was higher in the central retina, as observed in vehicle-treated animals. This topography did not reflect the sectoral loss of Brn3a+RGCs shown in Figures 1, and 2.

In intact retinas, PMCs/MdMs were few and scattered throughout the RGCL (17.5±6.2 PMCs/MdMs (n=4) Figure 5C’, I). After OHT, their number increased significantly at 4 and 15 days (1142±639 PMCs/MdMs (n=6) (p=0.0095, Mann-Whitney test) and 3530±1091 PMCs (n=6) (p=0.0095, Mann-Whitney test), respectively. Similar to Iba1+MCs, the increase in PMCs/MdMs was significantly lower in minocycline-treated retinas at both 4 (279±137 (n=5); (p=0.0043, Mann-Whitney test) and 15 days (1317±704 (n=6); (p=0.0043, Mann-Whitney test). Interestingly, the distribution of PMCs/MdMs followed the sectorial pattern of RGC death, which was particularly evident in the neighbourhood maps of vehicle-treated retinas (Fig. 5E'-G', I).

## DISCUSSION

In this paper, we show for the first time in a mouse model of glaucomatous injury induced by IOP elevation the progression of RGC death, its relationship with the expression of a-Casp3 and the dynamics of both microglial and phagocytic microglial cells, and the effect of minocycline treatment on RGC survival, caspase-3 activation, and microglial behaviour. Our work shows that RGC commitment to death occurs earlier than expected, a death that is not rescued by the action of minocycline in inhibiting microglial activation or caspase 3.

Double immunodetection of RGCs and a-Casp3 allows for a more precise determination of the therapeutic window for RGC protection because the activation of caspase 3 precedes the statistical loss of RGCs. Indeed, RGC death becomes statistically significant at 7 days (33.3% loss) while in the same retinas the number of a-Casp3+cells increased significantly already at 3 days peaking at 4 days. Brn3a is a marker of viability in adult RGCs [26, 56], while a-Casp3 is a marker of apoptosis. In the hypertensive retinas, we found a-Casp3+Brn3a+ RGCs, which are RGCs that are in the early stages of apoptosis, and a-Casp3+cells that are Brn3a negative, which are RGCs that are further into the apoptotic process, or other types of Brn3a-negative RGCs, such as melanopsin or ON alpha RGCs [55, 60], or even amacrine cells displaced in the RGC layer, although it has previously been shown that displaced amacrine cells are not affected in this injury model [11, 61].

In this study we observed a significant increase in a-Casp3+ cells at 3 days after OHT induction, the first time point of study. This early increase of a-Casp3 after retinal injury has been previously documented in retinal extracts by western blotting at 3 days post-injury, but not earlier. [62] However, 3 days after OHT may be too late. Indeed, while the overexpression of a-Casp3 in the study by Lu’s et al. [62] was significant at day 3, they also observed an increase in TUNEL+cells in the RGCL as early as 24 hours, albeit not in the same lesion model. Furthermore, TUNEL staining is a marker of late apoptosis, compared to the activation of Casp3 that marks the early stages of apoptosis [63]. a-Casp3 upregulation is a common early event observed in several RGC degeneration models, such as retinal organotypic cultures [24], ischemia-reperfusion [64] or ONT or ONC [13, 26, 37, 65]. Therefore, neuroprotective therapies for RGC rescue will most likely need to be administered early after the insult, well before loss of Brn3a expression in the RGC population.

The results of this study highlight the need to implement tools for in vivo detection of incipient retinal degeneration in clinical practice. Interestingly, a new technology based on the in vivo detection of a-Casp3 expression in the retina, known as DARC (Detection of Apoptosing Retinal Cells), is currently being translated from preclinical models to patients as a predictive tool for glaucoma progression, as a-Casp3 expression is an event that occurs prior to fibre layer thinning. [66, 67]. Here in post-mortem samples and using topographic maps, we observed that at 4 and 5 days, before significant RGC loss, a-Casp3+cells were more abundant in sectors where RGC degeneration would be expected to be greater.

The loss of RGCs progresses at least up to 30 days, and at this time the expression of a-Casp3 is still higher than in control retinas, although to a lesser extent than at early time points. This long-term expression of a-Casp3 in the RGCL has also been reported in the microbead-induced OHT model [68] and in genetic mouse models of glaucoma [69, 70]. Furthermore, the course of a-Casp3 in our OHT model is very similar to that reported after ONC or ONT [26]. In ONT or ONC models, RGC loss is diffuse and has two phases: a quick phase, when most of the RGCs die ( ?15% loss at 3 days, ?38% at 5 days, ?62% at 7 days, ?78% at 14 days) and a slow phase that lasts up to 90 days (?94% of loss) [26, 71], and, as reported for OHT, the expression of a-Casp3 is also transient, being significant at 24-48 hours after the injury peaking at 4 days and declining thereafter [26].

Minocycline has been tested as a neuroprotectant because it at least partially inhibits microglia, thereby reducing inflammation, and caspase 3, thereby impairing apoptosis [35, 37-44, 72]. As a result, minocycline has shown neuroprotective properties for RGCs and preservation of axonal integrity in injury models with an inflammatory and apoptotic component such as chronic OHT [34, 73], ischemia-reperfusion [68, 74] and photic injury [49, 72]. However, although minocycline is neuroprotective in chronic models of OHT, in the present study it did not reduce RGC death, possibly because the course of RGC death is faster in our model. Although there is no direct correlation between the death of RGCs and the peak of OHT or the area under the elevated IOP curve [11], other mouse models of OHT showed no significant RGC death after 8 weeks of a mean IOP elevation of 25 mmHG [75]. This suggests that in our model, which produces a higher IOP, there may be an ischemic component.

Although there is a significant decrease in a-Casp3+ cells, MCs and PMCs/MdMs in minocycline-treated animals at 4 and 15 days, none of these changes rescued RGCs in our OHT model. These findings may indicate that other caspase-3 independent programmed or non-programmed death pathways may play a role in the death process of RGCs after OHT. In models of elevated IOP, necroptosis, which occurs before apoptosis [76], pyroptosis, oxytosis/ferroptosis, and parthanatosis [69, 70, 77-79] have previously been shown to simultaneously contribute to inflammation and retinal damage as early as 24 hours after injury induction. Furthermore, Dvoriantchikova et al (2022) [79] showed high activity of the TNF pathway in the early stages of retinal injury induction by acute IOP elevation leading to early activation of necroptosis while significant activity of other programmed death pathways appears later, suggesting that glutamate and ferrous iron may be key players simultaneously triggering at least necroptosis, oxytosis/ferroptosis and parthanatosis in RGCs. Moreover, these results also suggest that microglial cells do not play a direct role in RGC death (up to 15 days), as has been shown after optic nerve axotomy [77]. However, we cannot know whether these changes have resulted in a functional improvement of the surviving RGCs.

Interestingly, inhibition of caspase3 with the irreversible caspase3 inhibitor ZDEVD_fmk results in increased survival of Brn3a+RGCs after optic nerve axotomy [26] a neuroprotection that is similar to that documented after administration of brain-derived neurotrophic factor (BDNF). The enhanced RGC rescue reported by Sánchez-Migallón et al. (2016) [26] differs from our results here. This may be due to the model and the different drug used or the fact that the ZDEVD_fmk was administered intravitreally whereas we administered minocycline systemically.

After OHT, Iba1+MCs increase 6- and 7-fold from baseline at 4 and 15 days, respectively, and this increase is controlled by minocycline, although not completely (4- and 4-fold, respectively), in agreement with previous reports [37, 38, 40, 74, 78, 80]. The treatment is also effective in reducing the number of PMCs/MdMs (from 65 and 202-fold in vehicles, to 16 and 75-fold in minocycline-treated retinas at 4 and 15 days respectively).

Regardless of treatment, Iba1+MCs are found evenly distributed throughout the retina, rather than in sectors with higher RGC death, but not so PMCs/MdMs, which are located in the sectors of higher RGC death. In contrast to the density distribution of a-Casp3+cells, the sectorial distribution of PMCs/MdMs was clearer at longer time points than at early time points. Thus, commitment to death and RGC clearance from the tissue are two temporally separated events. Therefore, RGC survival should be assessed using phenotypic markers of viability rather than tracers that are only removed from the tissue by phagocytosis. This is particularly important when using neuroprotective therapies that target microglia, as reduced phagocytosis would result in more traced RGCs in the tissue, which may not necessarily be alive [29, 33].

This study has several limitations. Because we used Iba1 to detect microglial cells, and this marker is also expressed in activated macrophages, we cannot exclude the effect of blood-derived infiltrating MdMs in OHT injury, and they may be important contributors to retinal inflammation. To detect RGCs, we used Brn3a, a viability marker that is expressed by most mouse RGCs and allows accurate assessment of RGC loss [81]. However, this marker is not expressed in some RGC subtypes, such as melanopsin+ RGCs or ON-alpha RGCs [60], so we do not know whether these RGCs are also susceptible to OHT. It is important to note that this model of IOP elevation may include an ischaemic insult that may contribute to or enhance some other death pathways, such as necroptosis. Another limitation of this work is the lack of functional assessment of the retina. A functional analysis would have been very interesting as it would have shown whether minocycline, despite not rescuing RGCs, improved retinal function [80]. Finally, this study was carried out in male mice, so further research is needed to prove that the same results can be obtained in female mice.

In conclusion, RGC death commitment starts very early after IOP elevation and therefore neuroprotective therapies should be applied well before significant anatomical or functional loss. Finally, our data indicate that in this OHT model, apoptosis is not the only death pathway activated in RGCs and, importantly, microglial cells are not a major contributor to RGC death.

## Supplementary Materials

The Supplementary data can be found online at: www.aginganddisease.org/EN/10.14336/AD.2024.0224
