# Supplementary material for: Minocycline Administration Does Not Have an Effect on Retinal Ganglion Cell Survival in a Murine Model of Ocular Hypertension
Source: Aging Dis. 2024 Oct 1;15(5):2241–54. doi: 10.14336/AD.2024.0224 (PMC11346395; doi:10.14336/AD.2024.0224)
Supplement: Supplementary file 1 — The Supplementary data can be found online at: www.aginganddisease.org/EN/10.14336/AD.2024.0224 [file AD-15-5-2241-s.pdf]

SUPPLEMENTARY DATA

Minocycline Administration Does Not Have an Effect on Retinal Ganglion Cell Survival in a Murine Model of Ocular Hypertension

María del Cielo Sánchez-Migallón, Johnny Di Pierdomenico, Alejandro Gallego-Ortega, Diego García-Ayuso, Manuel Vidal-Sanz, Marta Agudo-Barriuso, Francisco J. Valiente-Soriano

© 2024. Sánchez-Migallón MC et al. Published online at http://www.aginganddisease.org/EN/10.14336/AD.2024.0224

SUPPLEMENTARY DATA

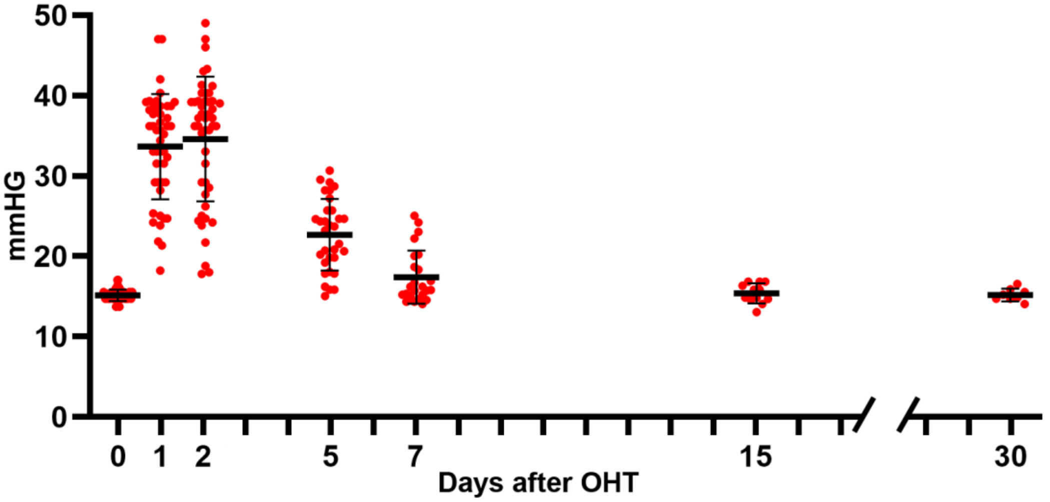

Supplementary Figure 1. Scatter-plot IOP evolution after OHT induction. X, Y scatter plot (time, IOP) showing IOP values (mmHg) of each sample (red dots) and mean± SD (black) in experimental left eyes before and 1, 2, 5, 7, 15 and 30 days after OHT induction (0-, 1- and 2-days n=48, 5 days n=32, 7 days n=24, 15 days n=16, 30 days n=8).

© 2024. Sánchez-Migallón MC et al. Published online at http://www.aginganddisease.org/EN/10.14336/AD.2024.0224
